# The Impact of Threatening Facial Expressions on Negotiation: An Eye‐Tracking Study

**DOI:** 10.1002/brb3.70461

**Published:** 2025-04-07

**Authors:** Mohammad Hossein Majidi, Khatereh Borhani

**Affiliations:** ^1^ Institute for Cognitive and Brain Sciences Shahid Beheshti University Tehran Iran

**Keywords:** autistic traits, eye‐tracking, facial expressions, social decision‐making, threat, Ultimatum Game

## Abstract

**Introduction:**

Facial expressions play a crucial role in social interactions, influencing trust, and decision‐making. In negotiations, threatening expressions may convey dominance or hostility, potentially reducing cooperation. This study explores how threatening facial expressions and autistic traits (ATs) affect social decision‐making in the Ultimatum Game (UG), focusing on their main effects on UG offers.

**Method:**

Fifty adults participated in the study. A Linear Mixed Model (LMM) was conducted to analyze the main effects of threat level and ATs on UG proposals. In addition, eye‐tracking technology was used to investigate participants' visual attention toward different facial areas.

**Results:**

The results revealed a significant main effect of threatening facial expressions, as participants made lower offers in response to high‐threat faces. However, ATs did not show a significant main effect on UG proposals. Eye‐tracking data showed that participants focused more on the eyes of high‐threat faces compared to low‐threat faces.

**Conclusion:**

These findings support the Emotion‐as‐Social‐Information (EASI) model, suggesting that emotional expressions, particularly threatening ones, influence negotiation behavior. The study enhances understanding of how facial cues and individual differences in ATs affect cooperation and decision‐making in social interactions.

## Graphical abstract

Threatening facial expressions negatively impacted negotiation outcomes in the Ultimatum Game, while autistic traits had no significant effect. Eye‐tracking data showed participants focused primarily on the eye region of high‐threat faces, highlighting its vital role in shaping social decision‐making compared to other facial areas.

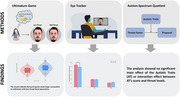

## 1 Introduction

Emotions serve as a powerful medium for communicating intentions, desires, and social signals, shaping interactions across various domains of life (Keltner and Haidt 1999). In fact, through emotions, individuals can express a wide range of signals, such as approval, disapproval, dominance, submission, or cooperation, which, in turn, can influence how others perceive and respond to them (Reis and Collins 2004; Van Kleef 2010). In addition, it has frequently been stated (e.g., Zebrowitz and Montepare 2008) that one of the most effective and immediate ways emotions are expressed is through facial expressions, which enable rapid, nonverbal communication. Indeed, the human face has the unique ability to convey both simple emotions, such as happiness and sadness (see, e.g. Ekman and Friesen, 1978), and more complex expressions, such as mistrust or threat (Oosterhof and Todorov 2008). These facial expressions provide essential information in interactions, enabling others to infer intentions and desires even in the absence of verbal communication (see Todorov et al. 2015).

Among the most complex and socially significant facial expressions are threat‐related expressions which signal salient emotional states and information concerning harmful intentions (Osterhof and Todorov 2008). Threat‐related expressions play a crucial role in various social contexts, particularly in negotiations, where they can signal dissatisfaction, assert power, or influence the perception of trustworthiness (Lelieveld et al. 2011; Brett and Thompson 2016; Adam and Brett 2015). For instance, a negotiator displaying threatening signals might be perceived as holding a stronger position, potentially leading to greater concessions from their counterpart (Geniole et al. 2019). However, the effects of expressing threat‐related expressions are not always straightforward. Research also suggests that such expressions can sometimes lead to adverse outcomes, depending on the context (see Van Kleef and Côté 2007 or Adam and Brett 2015). We will return to this point later in this article.

Furthermore, it is worth noting that the processing of emotional expressions is not uniform across individuals. In other words, multiple factors can influence how people interpret and react to social signals and emotional states conveyed by facial expressions. One key factor affecting social and emotional recognition is autistic traits (ATs; Couture et al. 2010; d'Arc et al. 2016; Khalil et al. 2018). In this study, we examined how threat in facial expressions influences decision‐making during negotiations and how ATs impact this process.

### 1.1 The Emotion as Social Information (EASI) Model

Van Kleef (2009), in his seminal work, introduced the EASI model, which explains how emotional expressions, at interpersonal level, influence observers' behavior through two primary mechanisms: *inferential processes* and *affective reactions*. Inferential processes enable individuals to interpret emotional expressions as signals about the expresser's goals, intentions, or boundaries, which can, in turn, shape their decision‐making. Specifically, in negotiation settings, individuals may make concessions in response to an opponent's display of anger (Pietroni et al. 2008). Affective reactions, on the other hand, involve direct emotional responses to others expressions, often through emotional contagion. In competitive settings like negotiation, perceiving anger in counterparts as a sign of threat—compared to happy or neutral counterparts—leads to the perception that they have higher expectations, thereby prompting greater concessions (Van Kleef et al. 2004a). Furthermore, the EASI model posits that the dominance of these processes depends on a range of factors, such as power dynamics or time pressure, which serve as moderators determining whether inferential or affective responses shape observers' behavior.

Given the significant impact of these interpersonal effects of emotional states in various contexts, particularly in economic negotiations, it is essential to examine how these expressions influence the negotiation process. In this study, we aimed to investigate how participants perceive complex facial expressions in negotiation scenarios. More specifically, we sought to determine whether inferential processes or affective reactions play a dominant role in shaping participants' behavior in such contexts. One effective approach to exploring this issue is through the *Ultimatum Game (UG)*. In the next section, we will review recent findings on the effects of counterparts' social and emotional states on social decisions using this paradigm.

### 1.2 Negotiation Using Ultimatum Game (UG)

As stated above, the UG is a widely accepted paradigm in behavioral economics and game theory for examining negotiation, cooperation, and the psychological factors (e.g., emotion expression) that influence decision‐making in social and economic interactions (Sanfey 2007). In this game, two players are involved: a proposer and a responder (Güth et al. 1982). The proposer is given a sum of money and is required to propose how to divide it with the responder. The responder, in turn, can either accept or reject the offer. If the responder accepts, both players receive their respective shares as proposed. However, if the responder rejects, neither player receives anything.

Interestingly, empirical results from UG studies often deviate from the predictions of *rational choice theory*. This theory suggests that the proposer should offer the least possible share to the responder, and the responder should accept any offer, no matter how small (Von Neumann and Morgenstern 1947). In contrast, a significant body of research has shown that proposers frequently tend to offer more than the minimum amount, and responders often reject unfair offers, even at the cost of receiving nothing (e.g., Nowak et al. 2000 or Mussel et al. 2013). This divergence from rational choice theory underscores the importance of social and psychological factors in shaping economic decision‐making.

Among these factors, threat‐related emotions such as anger have been found to significantly influence the outcomes of the UG. To clarify, some studies suggest that when proposers encounter angry responders, they tend to modify their proposals to be more favorable, aiming to alleviate tension and ensure acceptance (Sinaceur and Tiedens 2006; Adam et al. 2010; Van Kleef et al. 2010; Geniole et al. 2019; Reed et al. 2014). However, other research indicates that the presence of anger may induce a sense of mistrust and disrupt cooperation, leading proposers to become less inclined to make concessions (Van Kleef et al. 2004a, 2004b; Van Kleef et al. 2006; Lelieveld et al. 2013; Adam and Shirako 2013; Adam and Brett 2015; Ferracci et al. 2021; Wang et al. 2023).

These conflicting findings highlight the complex role of threat‐related emotions in the UG setting. In fact, researchers increasingly believe that the impact of threatening signals may depend on contextual factors (see Adam and Brett 2015). For instance, Lelieveld et al. (2011) found that during negotiations, expressing anger specifically toward the offer prompted participants to utilize the emotional cues to evaluate the boundaries set by the counterparts. This resulted in a higher likelihood of making concessions. However, when anger was directed toward the participants themselves, it did not result in an increased willingness to make concessions.

This complexity is further compounded when threatening signals are conveyed via facial expressions. In the following section, we will discuss the role of facial expressions in shaping negotiation outcomes.

### 1.3 Facial Expressions and Trustworthiness

We naturally form first impressions from faces, even when explicitly warned against doing so (see Todorov et al. 2015). As stated earlier, facial expressions, in particular, serve as crucial social signals that convey emotions and influence interpersonal interactions (Zebrowitz and Montepare 2008). Given their importance, numerous studies have explored the role of facial expressions across various settings, particularly in the context of negotiation.

However, most research has focused on the impact of basic, explicit emotions (e.g., happiness or anger) on the social interactions. For instance, research by Campellone and Kring (2013) using iterated trust games demonstrated that anger significantly reduces trust, whereas happiness has little effect on cooperation. Expanding on this, Weiß et al. (2019) examined how affective emojis—those representing explicit emotions—impact social interactions in bargaining scenarios. Their findings revealed that real facial expressions exerted a stronger influence on decision‐making in the UG, encouraging cooperative behavior. In contrast, emojis did not significantly alter participants' acceptance rates or decisions, suggesting that they may not have the emotional depth and social weight of real facial expressions in guiding cooperation.

On the other hand, beyond basic emotions, the human face is capable of producing more complex and nuanced expressions. Indeed, it has been shown that specific facial features can signal a perceived threat and influence trustworthiness judgments. Characteristics such as a furrowed brow, narrowed eyes, and a tense mouth can collectively convey a sense of potential threat or distrust (Fox and Damjanovic 2006; Oosterhof and Todorov 2008; Dotsch and Todorov 2012). While some studies have demonstrated that individuals tend to focus predominantly on the eye region, revealing its significance in conveying threat (Calvo et al. 2018; Eisenbarth and Alpers 2011; Schurgin et al. 2014; Hermens et al. 2018; Biermann et al. 2021), other research suggests that the mouth area plays a significant role in the perception of trustworthiness (Calvo et al. 2018; Dzhelyova et al. 2012; Sutherland et al. 2015; Bylianto and Chan 2023). The discrepancy in findings suggests that various factors, such as the nature of the task, can influence how trustworthiness is perceived.

For example, Calvo et al. (2018) demonstrated that participants' gaze behavior varied according to the task. These researchers found that while processing happiness, participants focused more intently and for longer durations on the mouth, whereas during trustworthiness assessments, the eye region was more significant than the mouth. This finding suggests that the eye region may provide more valuable information when evaluating complex facial expressions such as trustworthiness. Interestingly, other studies have shown that trustworthiness judgments are influenced not only by individual facial cues but also by the interaction between different cues. For instance, participants relied on the congruence between eye gaze—characterized by inverted V‐shaped eyebrows—and a smiling mouth to assess trustworthiness (Hermens et al. 2018; Dzhelyova et al. 2012; Kaisler and Leder 2017).

However, the existing body of research on trustworthiness perception through facial expressions suffers from several limitations. Notably, the predominant use of neutral or happy facial expressions in previous studies may not fully capture the complexities of trust assessment in real‐world scenarios, where threat cues can significantly influence perceptions. Moreover, although eye‐tracking technology has been employed in facial expression studies, few studies have specifically examined how threatening facial expressions impact gaze patterns and trust evaluations. In fact, this technology allows us to identify the visual attention elements involved in perceiving facial expressions as threatening. Given that individuals often rely more on the eye region when assessing more complex facial expression such as trustworthiness (Calvo et al. 2018; Biermann et al. 2021), incorporating advanced eye‐tracking technology in studies of threatening facial expressions could offer valuable insights into how people process faces in high‐stakes contexts, such as negotiations or interpersonal conflict.

### 1.4 Autistic Traits and Their Influence on Cooperation

As highlighted in the overview, individuals process emotions differently, which becomes particularly important when interpreting complex and implicit emotional expressions. Indeed, studying social negotiations involving threat‐related information can be especially challenging when individuals exhibit varying levels of social and emotional comprehension. This is particularly evident when individuals demonstrate high levels of ATs. ATs refer to a spectrum of characteristics associated with *Autism Spectrum Disorder (ASD)* that can be present to varying degrees in the general population. These traits include difficulties in social interaction and challenges in emotion recognition (Khalil et al. 2018; Hase et al. 2023; Jameel et al. 2014; Woodcock et al. 2020; Ramachandra and Longacre 2022). While individuals with high levels of these traits may not meet the clinical criteria for an ASD diagnosis, they can still exhibit social and behavioral patterns that differ from those with lower levels of ATs (Baron‐Cohen et al. 2001; Landry and Chouinard 2019; Hooper et al. 2019). Therefore, understanding how these traits manifest in the general population is essential for exploring their impact on various aspects of behavior, including cooperation (e.g., Craig et al. 2017).

Cooperation in negotiation, from a game theory perspective, involves strategic interactions where parties seek mutually beneficial outcomes (Nash 1950). Previous research on whether individuals with *High Autistic Traits (HAT)* exhibit reduced cooperation compared to those with *Low Autistic Traits (LAT)* has yielded mixed results. Some studies suggest that HAT individuals struggle with cooperation, likely due to deficits in *Theory of Mind (ToM)* and social cognition (Liebal et al. 2008; Colombi et al. 2009). For example, Liebal et al. (2008) showed that autistic children performed worse in cooperative tasks with adults compared to neurotypical peers. However, other studies found no significant differences in cooperation between autistic and neurotypical individuals, especially in structured economic games like the *Prisoner's Dilemma Game* (*PDG*; Downs and Smith 2004; J. Li et al. 2014). Surprisingly, Craig et al. (2017) even reported that HAT individuals cooperated more effectively with AI agents than with neurotypical partners, suggesting a context‐dependent effect.

These discrepancies may stem from variations in task structure, severity of autism, sample characteristics, and definitions of cooperation (Peng et al. 2024; K. Li et al. 2024). Thus, while some research supports the idea that ATs impair cooperation in negotiation, others indicate that cooperation difficulties depend on social context and interaction partners.

### 1.5 The Present Study

Prior research has demonstrated that the effectiveness of emotional expressions in negotiations is highly dependent on contextual factors (e.g., Van Kleef et al. 2010). Specifically, expressions of anger may elicit different responses depending on whether the negotiation setting is cooperative, competitive, or balanced (Adam and Brett 2015). This underscores the importance of considering context when examining the role of emotions in negotiation.

Despite this, most studies have primarily focused on basic and explicit emotional expressions, such as anger or happiness. However, complex facial expressions, such as threatening expressions, are particularly relevant in negotiations, where subtle nonverbal cues can shape perceptions of trustworthiness and willingness to cooperate. Nevertheless, little is known about how such complex and implicit expressions influence decision‐making in these settings (for an exception, see Geniole et al. 2019).

In this study, we aimed to investigate the role of threatening facial expressions in cooperative negotiations. Specifically, we examined how facial expressions conveying different levels of threat influenced cooperative behavior. Using the UG paradigm, we assessed how participants responded to counterparts displaying high‐threat versus low‐threat facial expressions in a negotiation setting. We hypothesized that low‐threat responders would be perceived as more trustworthy, fostering higher cooperation and increased concessions. To test this, we manipulated neutral facial expressions to be perceived as either low or high in threat and examined their impact on decision‐making within a cooperative negotiation framework^1^.

In addition, we investigated eye movement patterns during the UG task to understand how participants visually processed threatening facial expressions. Since we employed threatening facial expressions, and prior research has shown that the eyes play a crucial role in processing complex facial emotions (Eisenbarth and Alpers 2011; Hermens et al. 2018; Calvo et al. 2018), we hypothesized that participants would allocate more attention to the eye region, rather than the mouth, when evaluating trustworthiness.

Finally, we explored the role of ATs in cooperation when participants encountered threatening faces. Using the *Autism Spectrum Quotient (AQ)* in a non‐clinical sample, we investigated whether ATs influenced cooperative behavior. Given the inconsistent findings in the literature regarding the impact of ATs on cooperation, this remained an open question.

## 2 Method

### 2.1 Participants

A total of 50 university students (*M* = 25; *SD* = 4.93; 27 males) voluntarily participated in the study without receiving any reimbursement. Participants were required to maintain a tracking ratio exceeding 75%, and consequently, three female participants were excluded from the analysis due to their tracking ratio falling below this threshold. In addition, all participants self‐reported having normal or corrected‐to‐normal vision. Written consent was obtained from all participants, and the study was approved by the local ethics committee at the authors' affiliated university.

### 2.2 Apparatus and Materials

#### 2.2.1 Stimuli

**FIGURE 1:**
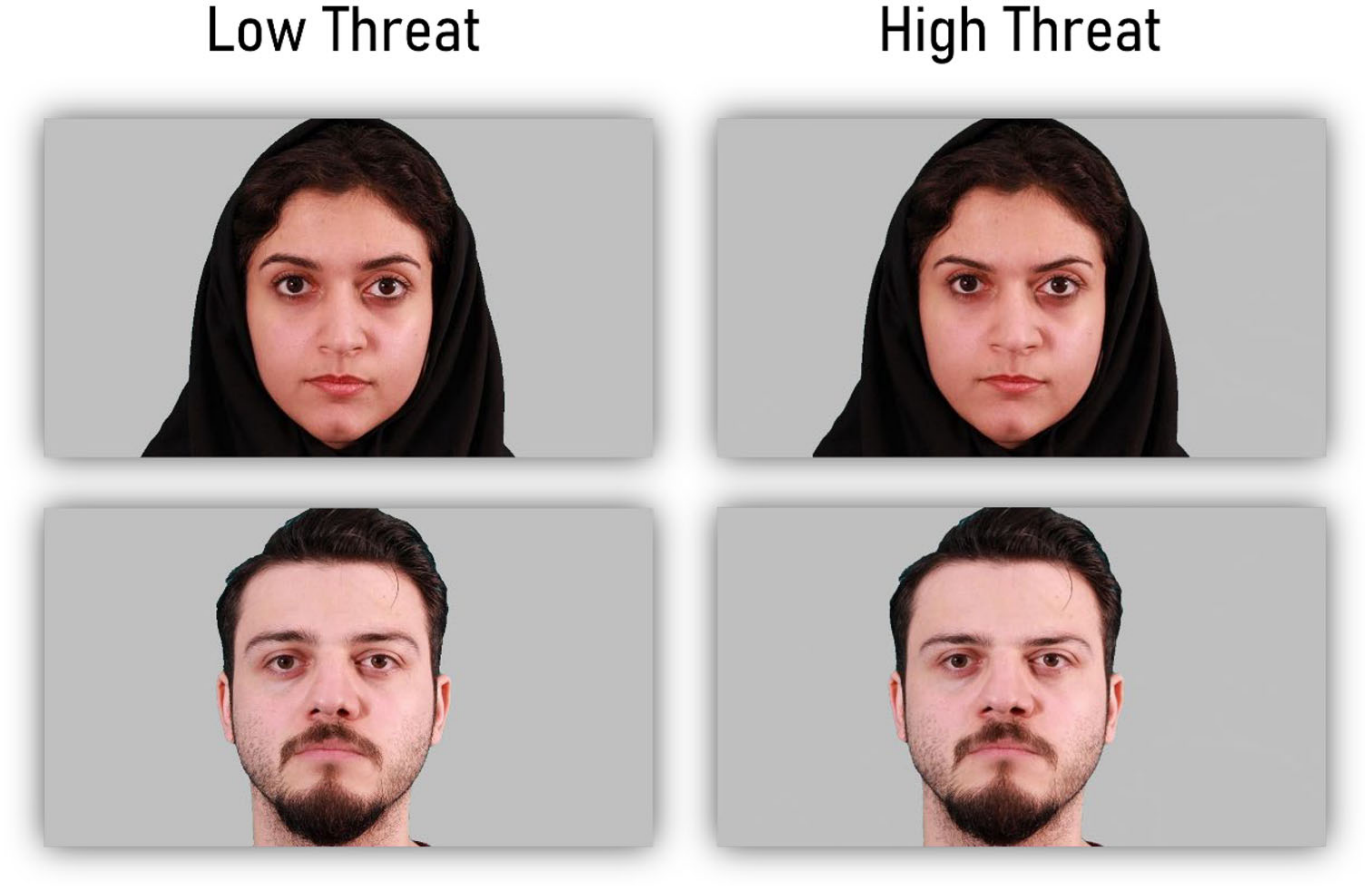
Examples of Facial Stimuli used in the Experimental task. Each participant was shown only one version of each face. Faces adapted from the Iranian Emotional Face Database.

From the *Iranian Emotional Face Database* (Heydari et al. 2023), we selected all facial images (including 25 male and 16 female faces) that displayed neutral expressions. In order to generate threatening facial images, we utilized computational models of facial threat (Oosterhof and Todorov 2008) to extract facial structure associated with threat. Subsequently, all facial images were adjusted along the threat dimension using PSYCHOMORPH software (version 6; Burt and Perrett 1995), resulting in faces depicting varying levels of threat. Consequently, two versions of each facial image were created, one depicting high‐threat and the other reflecting low‐threat (see Figure 1).

To evaluate the validity of our threat stimuli, we recruited a separate group of 32 participants (*M* = 25.42; *SD* = 3.52; 17 females). These participants were asked to rate the facial images based on their perceived level of “threat” using a 7‐point Likert scale (ranging from 1 = completely non‐threatening to 7 = highly threatening). In addition, we informed participants that the definition of “threat” encompasses any inclination or capability to cause harm to others, either physically or mentally (see Geniole et al. 2019). Since the ratings were not normally distributed, we employed the Wilcoxon test for analysis. The results demonstrated that the participants consistently rated high‐threat faces (*M* = 4.26, *SD* = 0.91) as significantly more threatening compared to low‐threat faces (*M* = 2.74, *SD* = 0.71), confirming the effectiveness of our manipulation across all facial images.

In the following step, with the aim of having an equal number of male and female faces, we intentionally selected 20 unique faces (comprising 10 male and 10 female faces) that exhibited the most distinct difference between the high‐ and low‐threat versions. Therefore, a collection of 40 facial images was prepared for the main study: 20 depicting high‐threat and 20 depicting low‐threat. This collection of 40 facial images was divided into two subsets, each comprising 20 unique faces. This allocation ensured that all 20 unique faces, including 10 with high‐threat expressions and 10 with low‐threat expressions, were present in each subset. Participants were exposed to only one of these two subsets during the study. In addition, we included an extra set of 12 unique facial images with neutral expressions (six male and six female faces) as filler stimuli, along with two additional neutral faces used in the practice phase (one male and one female face).

#### 2.2.2 Eye‐Tracking

**FIGURE 2:**
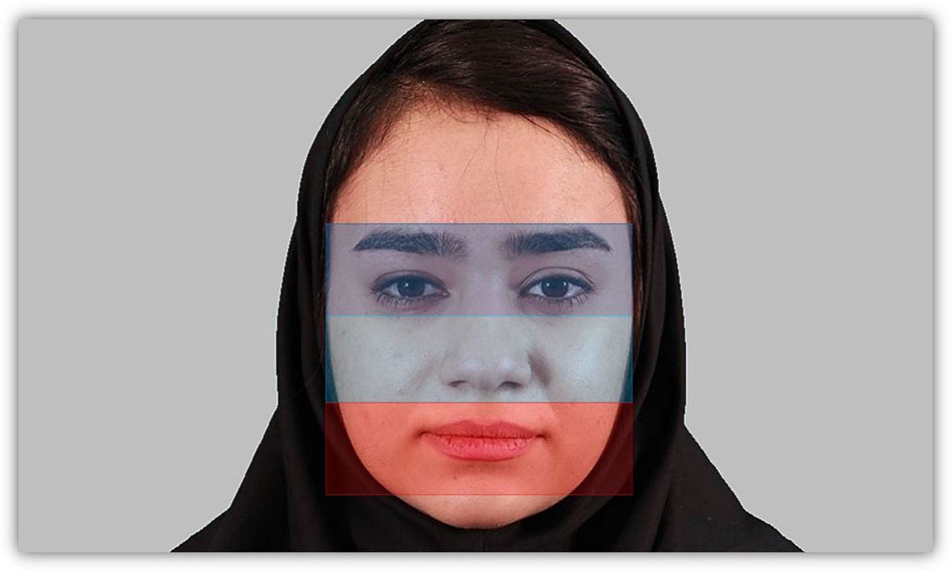
Illustration of Three facial areas of interest (AOI). The eye areas encompass the eyes, eyebrows, and the regions between them, while the nose and mouth areas encompass their respective regions and their surroundings. Each AOI was carefully delineated for each face individually.

For each participant, eye movement data were collected using a RED 250 Eye Tracker (SMI Technology, Germany). Data collection was conducted using iView software with a sampling rate of 250 Hz. The stimuli were presented on a 21‐inch monitor with a resolution of 1600×900 and arranged in a random order using the Experiment Premium Scientific Suite software. Subsequently, BeGaze software (version 3.4) was employed for data preprocessing. Notably, the data analysis focused on three distinct *areas of interest (AOIs)*: eyes, nose, and mouth (see Figure 2).

#### 2.2.3 The Autism‐Spectrum Quotient (AQ)

The AQ, developed by Baron‐Cohen et al. (2001), is a widely used self‐report questionnaire designed to measure ATs in the general population. Consisting of 50 items, it assesses behaviors and preferences associated with autism spectrum conditions across five domains: social skills, attention to detail, attention switching, communication, and imagination. Each item presents a statement where respondents indicate their degree of agreement on a Likert scale. Its application extends beyond clinical diagnosis to research exploring the broader impact of ATs on cognitive processes, social behaviors, and decision‐making across diverse populations (Austin 2005; Jameel et al. 2014; Zhao et al. 2020). The Farsi version of AQ‐50 demonstrates acceptable internal consistency (*α* = 0.79) and robust test‐retest reliability (*r* = 0.82, *p* < 0.05; Nejati Safa et al. 2003), making it an effective tool for identifying subclinical levels of ATs among Iranian individuals.

### 2.3 Task and Procedure

All participants engaged in a hypothetical scenario simulating the UG. The task was cooperatively designed to capture the main characteristics of real‐life negotiations (as described by Adam and Brett 2015). In this scenario, each participant assumed the role of a manager overseeing a clothing chain store. Their objective was to recruit a marketer for each of the store branches to assist in product sales. Consequently, all participants were informed that they would negotiate with a group of marketers to reach a collaboration agreement. Notably, they would only be presented with the marketers' faces, with no information about their professional backgrounds. The goal was to propose a percentage of sales profit to each marketer—a figure that would not only maintain branch profitability but also ensure marketer satisfaction. To enhance believability, participants were further informed that each marketer had a predetermined profit threshold; offers falling below this threshold would likely be rejected.

Participants were tested individually in a dimly lit room. Prior to eye tracker calibration, each participant received a comprehensive explanation of the scenario and its rules. Subsequently, participants were seated approximately 65 cm from the monitor. Using a standard 9‐point grid, the eye tracker was calibrated, resulting in a calibration error of less than 0.5° on either the horizontal or vertical axis for all participants. If necessary, recalibration of the eye tracker was performed until a precise and dependable estimate of the participants' eye positions was achieved.

**FIGURE 3:**
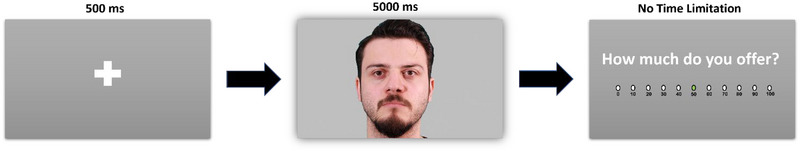
Sequence of events on each trial. The facial stimuli were presented in random order, with the constraint that only one version of each face (high or low threat) could be shown to each participant.

The experiment started with two practice trials to ensure participants were familiar with the procedures. Following the practice phase, if participants had no confusion about the experiment, the study phase initiated. In a series of 30 one‐shot UG trials, each participant assumed the role of a proposer. As shown in Figure 3, each trial began with a fixation cross displayed at the center of the screen for 500 ms, followed by a picture of a responder (the marketer) shown for 5000 ms. Afterward, participants were asked to select an offer from a set of predefined percentages (0%–100%) to determine the share of sales profit allocated to the proposer using the standard wireless mouse. Participants had no time limit for making decisions. Once participants provided an offer, one trial was completed, and the following trials were presented in the same sequence. It is worth noting that no feedback was provided after each trial. This approach was adopted to prevent the influence of previous outcomes on subsequent decisions, consistent with previous research (Rezlescu et al. 2012). Following the experimental section, participants completed an AQ questionnaire online. Finally, participants were thanked and informed about the purpose and content of the experiment.

### 2.4 Data Analysis

All data analysis was conducted using Python version 3.10.1 (Python Software Foundation, 2023). From the behavioral data, we computed the average offer made in response to high‐threat and low‐threat facial expressions for each participant. In order to account for individual variability and better model the hierarchical structure of the data, we conducted a *Linear Mixed Model (LMM)* analysis, treating threat level (high and low) and AQ score as fixed factors and a random effect for participants. To assess whether ATs moderates the effect of threat on UG offers, we compared a “full” model including the threat level × AQ score interaction to a “reduced” model containing only main effects. A *Likelihood Ratio Test* (*χ^2^* (1) = 1.40, *p* = 0.237) confirmed that the interaction term did not significantly improve model fit. Therefore, for interpretability, we proceeded with the main effects model, focusing on the effects of threat level and AQ score independently.

Eye‐tracking data underwent preprocessing using BeGaze software (version3.4, SensoMotoric Instruments, Teltow, Germany), with a specified minimum fixation duration of 100 ms. AOIs were specified for each face stimulus, comprising the eyes, nose, and mouth. The eye area was defined as a rectangle covering the eyes, eyebrows, and the space between them. In addition, separate rectangles were designated for the nose and mouth areas, encompassing their respective areas and nearby surroundings (see Figure 2). The *Normalized Dwell Time (NDT)* parameter was computed for each AOI and face stimulus and then averaged for each participant. *Dwell Time (DT)* was defined as the sum of durations from all fixations and saccades that hit the AOI in milliseconds (ms). The NDT, denoted as [ms/coverage], is computed by dividing the DT by the corresponding coverage AOI.

Moreover, as AQ did not significantly influence the behavioral data, we excluded it from the eye‐tracking analysis to maintain model parsimony and focus on the primary effects of threat level and AOIs. A LLM was conducted on NDT with threat levels (high and low) and AOIs (eyes, nose, and mouth) as fixed factors. In cases of significant interaction effects, Tukey's post hoc comparison was conducted.

## 3 Results

### 3.1 Behavioral Results

**FIGURE 4:**
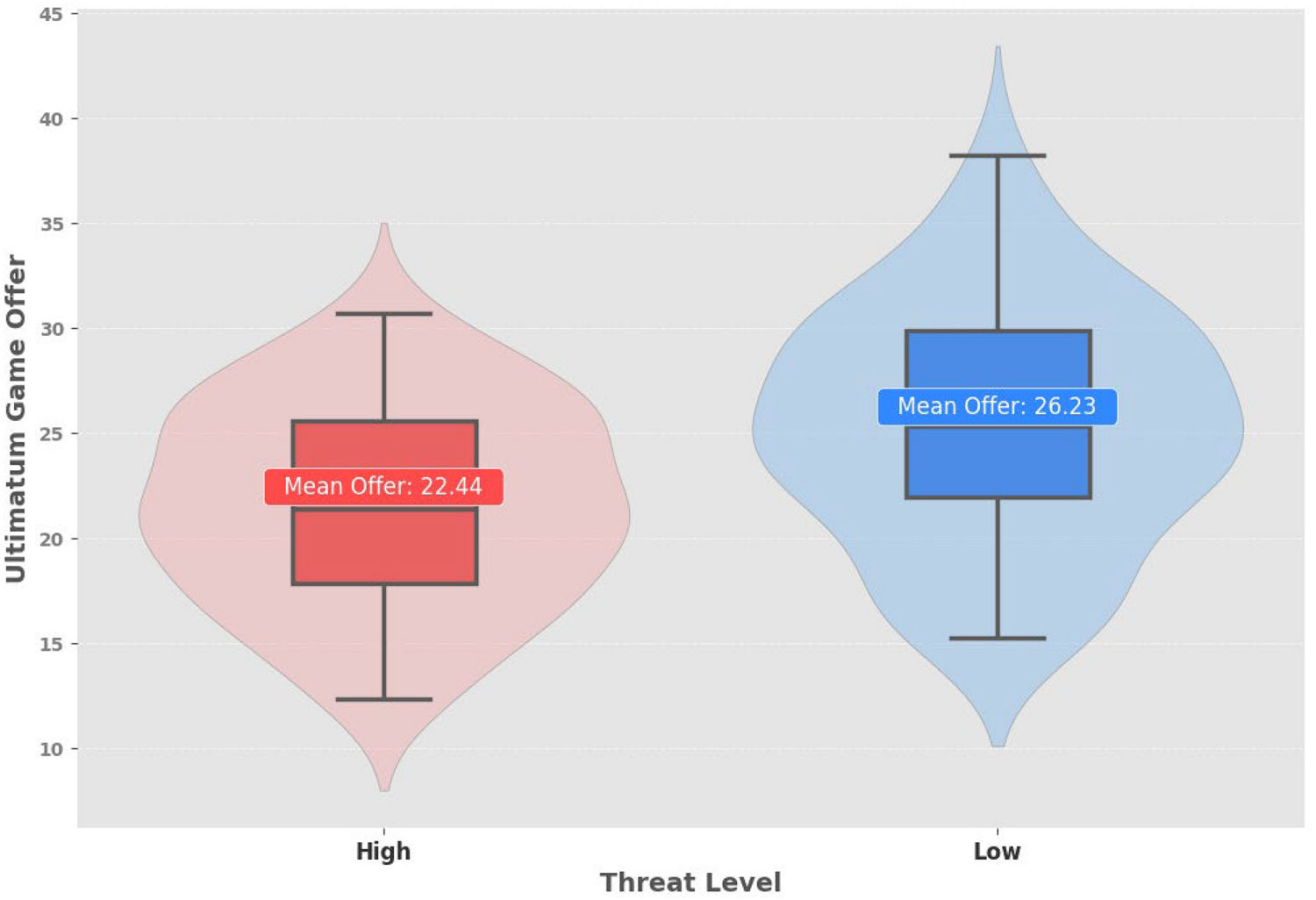
Main effect of threat on the proposer's offer. The violin‐box plot illustrates that participants offered greater concessions to individuals displaying low‐threat faces.

A LMM was conducted to examine the effect of threat level (high vs. low) and AQ score on participants' offers. The analysis revealed a significant main effect of threat level (*b* = 3.792, *SE* = 0.826, *z* = 4.592, *p* < 0.001), indicating that participants made higher offers in response to low‐threat faces (*M* = 26.23, *SD* = 13.56) compared to high‐threat faces (*M* = 22.44, *SD* = 14.32; see Figure 4). However, the main effect of AQ score was not significant (*b* = 0.202, *SE* = 0.373, *z* = 0.542, *p* = 0.588), suggesting that individual differences in AQ scores did not influence the offers made. These results indicate that threat perception significantly influences economic decision‐making, whereas ATs (AQ score) do not appear to play a role in offer adjustments.

### 3.2 Eye‐Tracking Results

A LMM was conducted to analyze the effects of threat level (high vs. low) and AOI (eyes, mouth, and nose) on NDT. The model included participants as a random effect and employed *Restricted Maximum Likelihood Estimation (REML)*.

**FIGURE 5:**
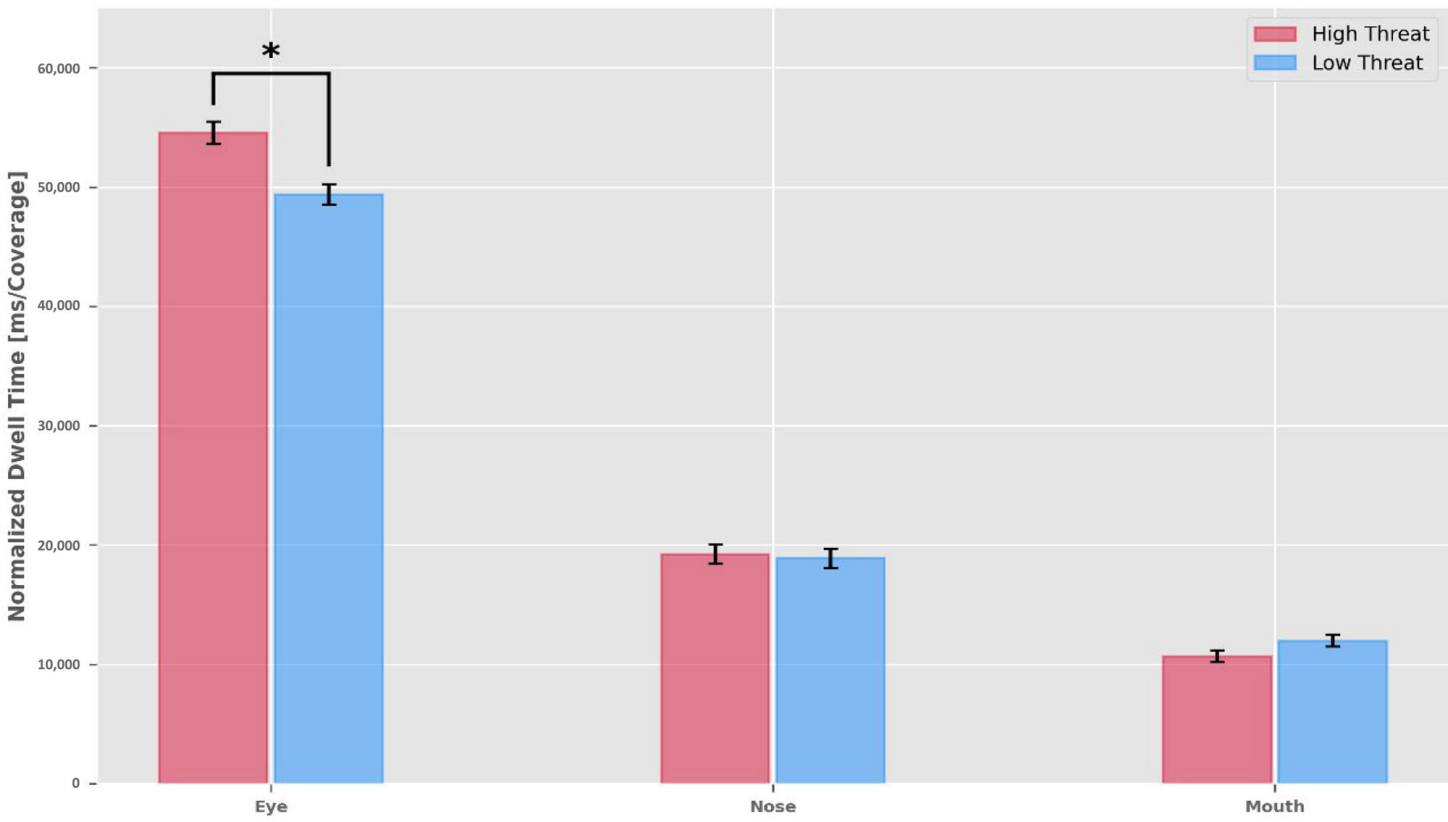
Mean NDT for each AOI (eye, nose, and mouth) on each face (high threat and low threat). The bar chart reveals that participants directed more attention to the eyes than to the nose or mouth areas of the face. The asterisk (*) indicates that the difference between high‐ and low‐threat faces in the eye region was significant at the *p* < 0.001 level. Vertical lines within the bars denote standard errors of the mean.

The analysis revealed a significant main effect of threat level (*χ^2^* (1) = 23.77, *p* < 0.001), indicating a shift in attention allocation when observing high‐ and low‐threat facial expressions. In addition, as shown in Figure 5, the main effect of AOI (*χ^2^* (2) = 1933.38, *p* < 0.001) indicated that participants exhibited varying NDT across different AOIs, with the eyes attracting the longest DTs, followed by the nose and mouth.

**FIGURE 6:**
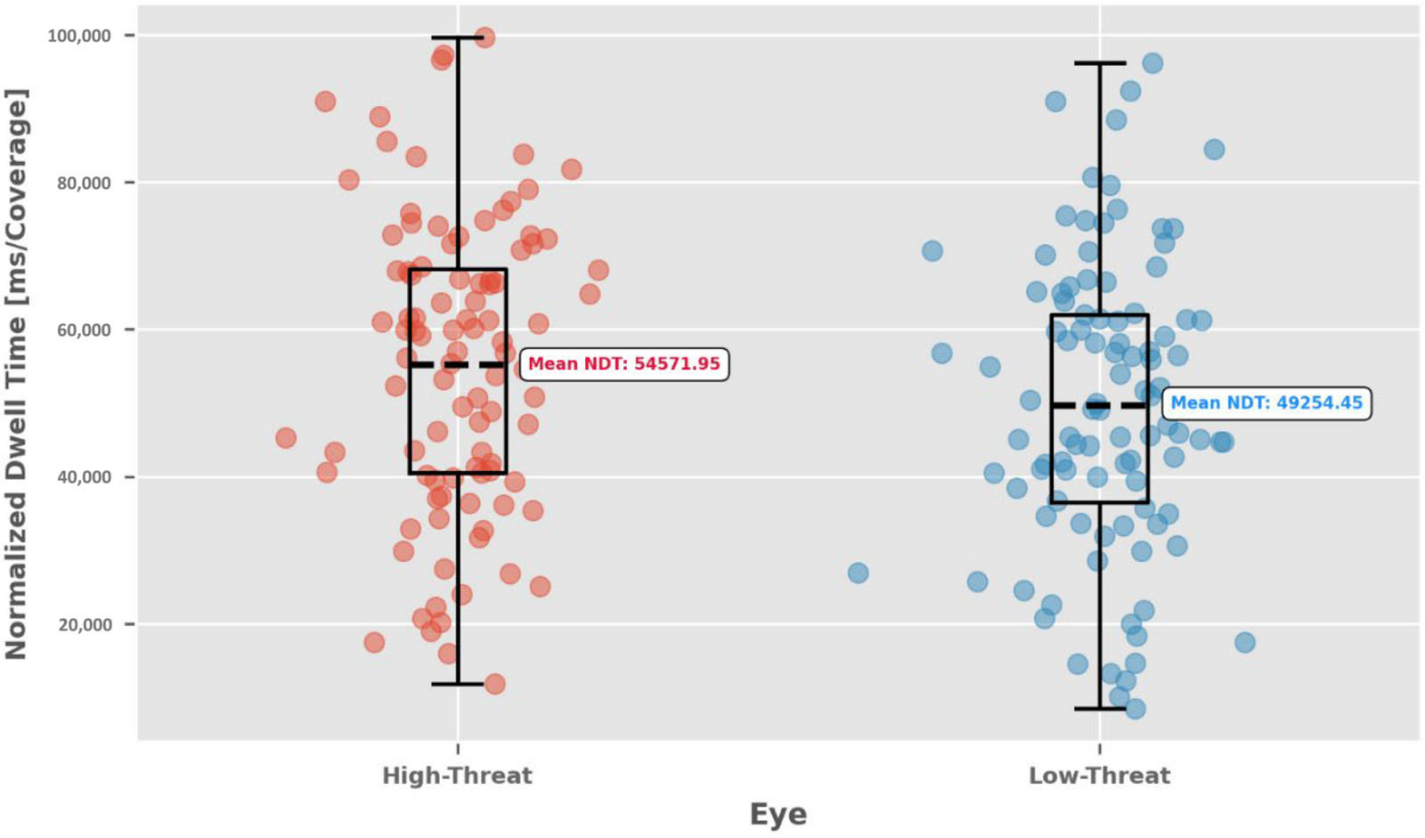
Normalized dwell time (NDT) on the eyes. The NDT on the eyes is higher for high‐threat faces than for low‐threat faces, indicating that participants may pay more attention to the eyes when assessing threatening faces.

Moreover, a significant interaction between threat level and AOI was found (*χ^2^* (2) = 20.28, *p* < 0.001). Tukey's post hoc tests revealed that this interaction was primarily driven by significant differences in NDT on the eye region between high‐threat (*M* = 54,571.95, *SD* = 19,915.86) and low‐threat faces (*M* = 49,254.45, *SD* = 17,844.39; see Figure 6). In contrast, no significant differences were observed in NDT for the mouth region (high‐threat: *M* = 10,667.24, *SD* = 10,479.57; low‐threat: *M* = 12,004.48, *SD* = 10,728.51) or the nose region (see Table 1). These findings suggest that threat perception selectively modulates attentional allocation to facial areas, particularly by enhancing attention to the eyes of high‐threat faces.

**TABLE 1:** Results of Tukey's post hoc analysis for NDT by AOI and threat factor.

Comparison	AOI	Estimate	*SE*	*t*	*p*
High versus low	Eyes	5158	1060	4.876	< 0.001
High versus low	Nose	325	1060	0.307	0.999
High versus low	Mouth	−1324	1060	−1.252	0.811

Abbreviations: MD = mean difference, SE = standard error.

## 4 Discussion

This study provides valuable insights into three major areas of research. First, it enhances our understanding of how complex facial emotions, especially threatening expressions, influence individuals’ decision‐making in cooperative negotiation settings. Second, it deepens our knowledge of the specific facial areas that are key to assessing trustworthiness, particularly in the context of threatening faces. Lastly, it illuminates the connection between ATs and cooperation in the general population. Each of these contributions is discussed in greater detail below.

### 4.1 Effect of the Threatening Facial Expressions in the Negotiations

Our study investigated the influence of embedded threatening cues in facial expressions on the cooperative behavior in the UG. The findings revealed that participants made larger concessions to individuals displaying low‐threat faces compared to those showing high‐threat faces. These findings are consistent with the EASI model (Van Kleef 2009), which posits two primary pathways through which social and emotional expressions influence perceivers: inferential and affective.

As previously discussed, the inferential pathway involves perceivers interpreting emotions as cues to the sender's intentions. In this context, perceiving a threat may signal potential conflict, prompting individuals to make larger concessions to prevent escalation. Conversely, the affective pathway suggests that exposure to threatening signals during negotiation evokes negative emotions, such as a desire for retaliation, leading to smaller concessions. According to the EASI model, negotiators' responses to a threatening counterpart depend on whether inferential cues or affective reactions dominate their decision‐making.

Moreover, contextual factors play a crucial role in determining which pathway predominates. For instance, research by Van Kleef et al. (2010) and Adam and Brett (2015) emphasizes that the negotiation context shapes how emotional expressions are perceived. Adam and Brett (2015) demonstrated that the social impact of expressing anger varies with the competitiveness of the negotiation setting. To clarify, in cooperative scenarios, such as business partnerships, expressing anger may evoke negative responses, as recipients might reciprocate with anger and resist concessions. Conversely, in balanced contexts where cooperation and competition coexist, anger might be interpreted as a display of power and high standards, potentially leading to more favorable concessions.

Considering these insights, our findings appear to align with previous research. In fact, given that our study was conducted within a cooperative framework, participants likely perceived threatening signals as socially inappropriate. This perception could have hindered cooperation and trust, increasing the likelihood of negotiation impasses. As a result, participants seemed to rely more on affective reactions than inferential reasoning. It is also worth noting that previous studies have established a strong link between unfair behavior and reciprocal anger and aggression (Barclay et al. 2005; Kennedy et al. 2004), suggesting that expressing threatening signals in cooperative scenarios can trigger intense negative emotional responses.

Taken together, our results demonstrate that in cooperative negotiations, where trust plays a vital role, the expression of threatening facial expressions possibly causes perceivers to infer that their counterparts are uncooperative and unreliable. As a result, perceivers become less willing to trust their counterparts, leading them to make smaller concessions. Nonetheless, our findings suggest that threatening facial expressions may negatively impact decision‐making in structured bargaining tasks, such as the UG. However, whether this effect extends to real‐world cooperative negotiations, which involve additional factors such as prior relationships, verbal communication, and strategic intent, remains an open question for future research.

### 4.2 Facial Areas in Trustworthiness Perception

In testing our second hypothesis, we examined how participants' eye movements were directed when judging threatening faces in the UG task. Given the cooperative nature of our UG setup, which emphasized trust and cooperation, we hypothesized that participants would focus on facial regions most relevant for perceiving trustworthiness, particularly the eyes. As expected, our results revealed that participants predominantly directed their gaze toward the eyes rather than the nose or mouth. In addition, Analysis of NDT on these AOIs showed that participants paid significantly more attention to the eyes of high‐threat faces compared to low‐threat faces. Although there was a tendency to focus more on the mouth of low‐threat faces, this difference was not statistically significant.

Previous evidence in this regard discussed the findings based on either facial expressions’ trustworthiness or facial expressions’ valence. Our findings align with previous research highlighting the critical role of the eyes in trustworthiness judgments. Several studies have consistently shown that the eyes play a crucial role in the assessments of complex facial expressions such as trustworthiness (Eisenbarth and Alpers 2011; Schurgin et al. 2014; Hermens et al. 2018; Biermann et al. 2021). In fact, this emphasis on the eyes can be attributed to their role in conveying social and emotional cues that are vital for assessing another person's intentions and reliability.

Conversely, other studies have underscored the importance of the mouth in trustworthiness judgments, particularly when facial expressions are depicting positive valence, such as smiling (Calvo et al. 2018; Dzhelyova et al. 2012; Sutherland et al. 2015; Bylianto and Chan 2023). The discrepancy between these findings and our results can be attributed to the nature of the facial expressions used. Studies emphasizing the role of the mouth in trustworthiness assessments typically involve simple, explicit happy faces, where a smiling mouth is a salient cue that naturally draws attention. However, in our study, we focused on complex facial expressions (i.e., threatening faces), where the mouth does not stand out as clearly as in happy expressions. As a result, participants may have relied more on the eyes, which provide crucial information about both threat and trustworthiness.

Moreover, Calvo et al. (2018) highlighted that the objective of the task significantly influences which facial areas are prioritized. Their research demonstrated that, while the mouth is crucial for processing happiness (as a simple facial emotion), the eyes are more critical when assessing trustworthiness (as a complex facial emotion). This supports our findings, where the cooperative and trust‐oriented context of the UG task naturally directed participants' attention toward the eyes of their counterparts, especially under perceived potential threat.

Generally, the increased focus on the eye area in high‐threat faces suggests that participants relied on this region to gauge trustworthiness. This is consistent with the idea that the eyes are key indicators of trustworthiness, particularly in situations involving potential social threats. Our participants likely perceived high‐threat faces as less trustworthy, leading to smaller concessions in the UG task.

### 4.3 Relationship Between Autistic Traits and Cooperative Behavior

The last aim that we wanted to explore was to evaluate the moderating role of ATs in influencing cooperation under social threat, specifically when participants encountered threatening faces in the UG task. Given the mixed results in the literature, we did not formulate a direct hypothesis. Our findings indicate that while threatening faces significantly affected proposals, ATs did not show a significant impact, neither directly nor through interaction with the threatening facial stimuli.

These findings contribute to the growing body of literature on the complex relationship between ATs and cooperation, suggesting that ATs do not necessarily predict reduced cooperative behavior in the UG. While previous research indicates that individuals with HATs may struggle with cooperation due to difficulties in ToM and cognitive empathy (J. Li et al. 2014), both essential for understanding others' intentions in strategic interactions, our results align with Ikuse et al. (2018), who found that ATs did not significantly affect social decision‐making in the UG, even in the presence of social cues like eye contact. This suggests that cooperation in structured economic games such as the UG may remain intact among individuals with various ATs, particularly in non‐clinical populations. Notably, J. Li et al. (2014) found that while individuals with HATs cooperated comparably to their neurotypical peers in strategic economic games (e.g., the UG and PDG), they exhibited difficulties in physical coordination tasks requiring real‐time interaction with a partner. This distinction indicates that cooperation challenges in individuals with HATs may be more pronounced in dynamic, real‐time social interactions rather than structured, turn‐based decision‐making.

Further supporting this distinction, Craig et al. (2017)​ investigated cooperation in autistic and non‐autistic individuals, demonstrating that ATs do not necessarily reduce cooperation but rather influence it depending on task demands. It seems that when social interactions require flexibility and ToM, individuals with HATs struggle (K. Li et al. 2024). However, in structured environments with clear rules, their cooperation remains intact or even improves.

Taken together, these findings suggest that the impact of ATs on cooperation is context‐dependent. While social threat influenced UG decision‐making, ATs did not significantly modulate this effect in our study. This is likely to align with the broader literature indicating that cooperation in structured bargaining tasks is less affected by ATs compared to dynamic, interactive cooperation.

### 4.4 Limitation

This study provides valuable insights into how threatening facial expressions influence negotiation behavior; however, several limitations should be acknowledged. First, although we validated the stimuli used in our task, we did not collect explicit trustworthiness ratings, making it unclear whether participants consistently perceived high‐threat faces as less trustworthy. Therefore, future studies could incorporate post‐task ratings of trustworthiness to validate this assumption while ensuring that such assessments do not introduce demand characteristics that might influence negotiation behavior.

Second, the sample size was relatively small, particularly for analyzing individual differences in ATs. While our study included 50 university students, this may not provide sufficient statistical power to detect subtle effects related to ATs. In fact, the null finding regarding the role of ATs in UG proposals does not necessarily indicate that ATs have no influence; rather, it could be due to limited sample variability or statistical power.

Third, the study used a one‐shot UG paradigm, limiting our ability to explore the dynamics of negotiation over time. While this approach effectively isolates the immediate impact of threatening facial expressions, it does not allow for strategic adjustments or the development of trust, which are essential features of real‐world negotiations. Future research could use iterated UG paradigms or multi‐round bargaining tasks to assess whether the effects of threatening facial expressions persist across repeated interactions.

Another limitation of our study is that response time was not recorded as part of our analysis. This could have provided additional insights into cognitive processing during decision‐making. We followed prior studies in this field, in which the time window for analyzing eye movements was fixed rather than based on decision RTs (e.g., Ferracci et al. 2021; Geniole et al. 2019; Golbabaei and Borhani 2024; Rezlescu et al. 2012; Mussel et al. 2013). While some studies imposed a response time limit, others, like Rezlescu et al. (2012), did not and also excluded RT from their analysis. Future research should consider incorporating RT alongside eye‐tracking data to better understand the relationship between gaze behavior and decision‐making.

Finally, our study relied on static images to manipulate threat perception, whereas in real‐world negotiations, facial expressions are dynamic and change throughout the interaction. The use of video‐based stimuli or virtual reality paradigms in future research could provide a more ecologically valid approach to studying how facial threat influences negotiation outcomes.

In sum, while this study advances our understanding of how threatening facial expressions influence negotiation behavior, addressing these limitations in future research will help refine and expand upon these findings.

## 5 Conclusion

In conclusion, this study sheds light on the complex interplay between threatening facial expressions, social decision‐making, and ATs. The findings suggest that while threatening faces significantly influence decision‐making in the UG task, ATs do not significantly impact this process in a non‐clinical population. This study supports previous research indicating that negotiation context matters; specifically, in cooperative settings, threatening expressions can hinder collaboration. The eye‐tracking results further reveal that participants allocate more attention to the eyes of high‐threat faces, highlighting the critical role of eyes in assessing complex facial expressions such as trustworthiness. These insights contribute to a nuanced understanding of how complex facial expressions and individual differences influence the negotiation process and cooperation. Future research should explore these relationships with more varied methodologies and broader participant samples to deepen our understanding of these intricate processes.

## Author Contributions

**Mohammad Hossein Majidi**: data curation, software, visualization, writing–original draft, formal analysis, writing–review and editing, methodology. **Khatereh Borhani**: writing‐original draft, writing–review and editing, supervision, conceptualization, methodology, formal analysis, project administration.

## Ethics Statement

This study received ethical approval from the Bioethics Research Committee of Shahid Beheshti University, Tehran, Iran.

## Conflicts of Interest

The authors declare no conflicts of interest.

### Peer Review

The peer review history for this article is available at https://publons.com/publon/10.1002/brb3.70461.

## Data Availability

The data that support the findings of this study are available on request from the corresponding author.

## Endnotes

1 Generally speaking, a cooperative negotiation is a situation such as starting a business, where individuals are inclined to trust and collaborate with each other (see Adam and Brett 2015).
